# Data of ERPs and spectral alpha power when attention is engaged on visual or verbal/auditory imagery

**DOI:** 10.1016/j.dib.2016.03.049

**Published:** 2016-03-18

**Authors:** Mario Villena-González, Vladimir López, Eugenio Rodríguez

**Affiliations:** Escuela de Psicología, Pontificia Universidad Católica de Chile, Santiago CP: 7820436, Chile

**Keywords:** Attention, Mental imagery, ERP, Alpha power

## Abstract

This article provides data from statistical analysis of event-related brain potentials (ERPs) and spectral power from 20 participants during three attentional conditions. Specifically, P1, N1 and P300 amplitude of ERP were compared when participant׳s attention was oriented to an external task, to a visual imagery and to an inner speech. The spectral power from alpha band was also compared in these three attentional conditions. These data are related to the research article where sensory processing of external information was compared during these three conditions entitled “Orienting attention to visual or verbal/auditory imagery differentially impairs the processing of visual stimuli” (Villena-Gonzalez et al., 2016) [1].

**Specifications Table**

**Table:** 

Subject area	*Cognitive neuroscience*
More specific subject area	*Attention*, *Mental imagery*, *Mind wandering*
Type of data	*Text file*, *graph*, *figure*
How data was acquired	*Electroencephalogram* (*EEG*) *Biosemi active two 64 electrodes*.
Data format	*Analyzed*
Experimental factors	*Continuous EEG data was resampled to 512* *Hz, re-referenced to mastoids, filtered between 0.5 and 30 Hz, epoched and averaged to calculate Event-related potential (ERP). For spectral analyses, same processing was carried out except for the down-sampling to 1024 Hz and the band-pass filter between 0.5 and 100* *Hz.*
Experimental features	*ERP was calculated to a probe stimulus during three conditions; an external task, a visual imagery and an inner speech*. *Free*-*stimulus time windows for spectral analyses were obtained for each of the three conditions*.
Data source location	Escuela de Psicología, Pontificia Universidad Católica de Chile, Santiago, Chile
Data accessibility	*All data are provided in this article*

**Value of the data**

• Data from early ERP׳s components during 3 conditions are shown here using 3 different ways of amplitude measurement known as peak-to-peak amplitude, local peak and mean amplitude.
• These data could be useful to complement the discussion about different ERP amplitude measurements, and to encourage further researchers to explain in detail the measurement used which is important for the replication of data.
• The data provided here may inform many researchers that are investigating attention allocation using early components of ERPs and alpha band modulations.
• Data show P300 component during external task condition while it was absent during inward attention.
• These data could be compared to other data related to internal/external attention allocation for further insight about this topic.

## 1 Data

**Fig. 1:**
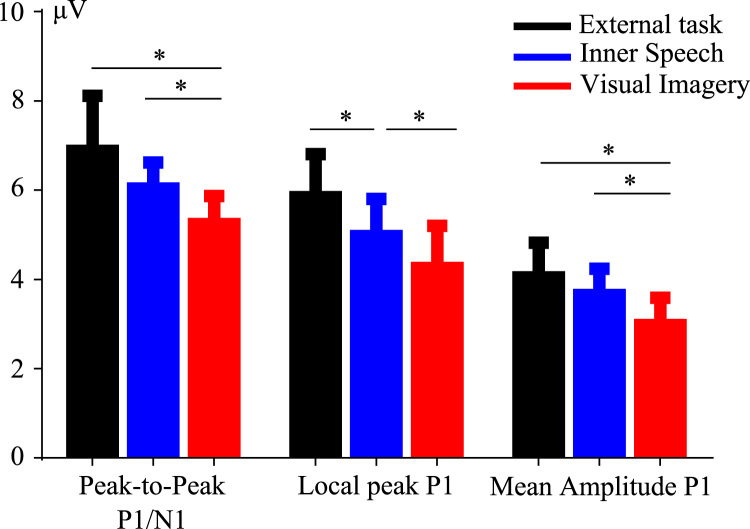
Amplitude of P1 for each condition computed by three different methods. P1 amplitude was computed by peak-to-peak, local peak and mean amplitude method. The same trend can be observed regardless of method, and the difference between both inward attention conditions is maintained.

**Fig. 2:**
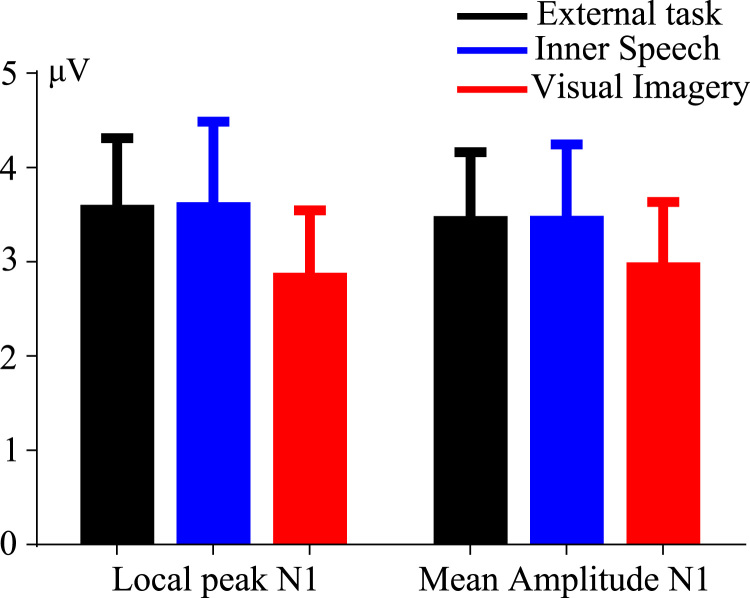
Local peak amplitude and mean amplitude of N1 component. N1 amplitude was computed by two different methods showing the same trend.

**Fig. 3:**
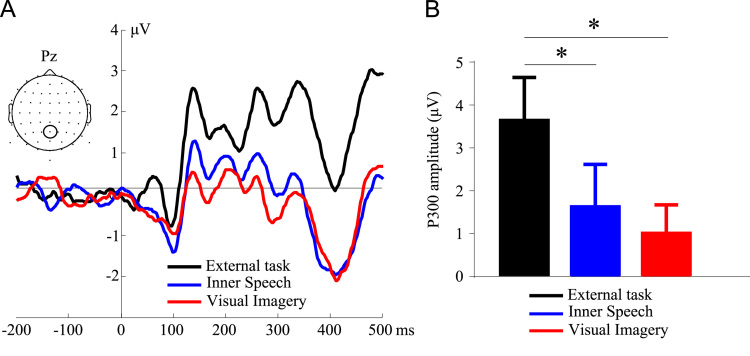
ERP waveform and P300 mean amplitude. (A) Grand average of the ERP waveform at Pz electrodes for the three conditions. (B) Mean and standard error of P300 amplitude for the three conditions. (*p<0.05).

**Fig. 4:**
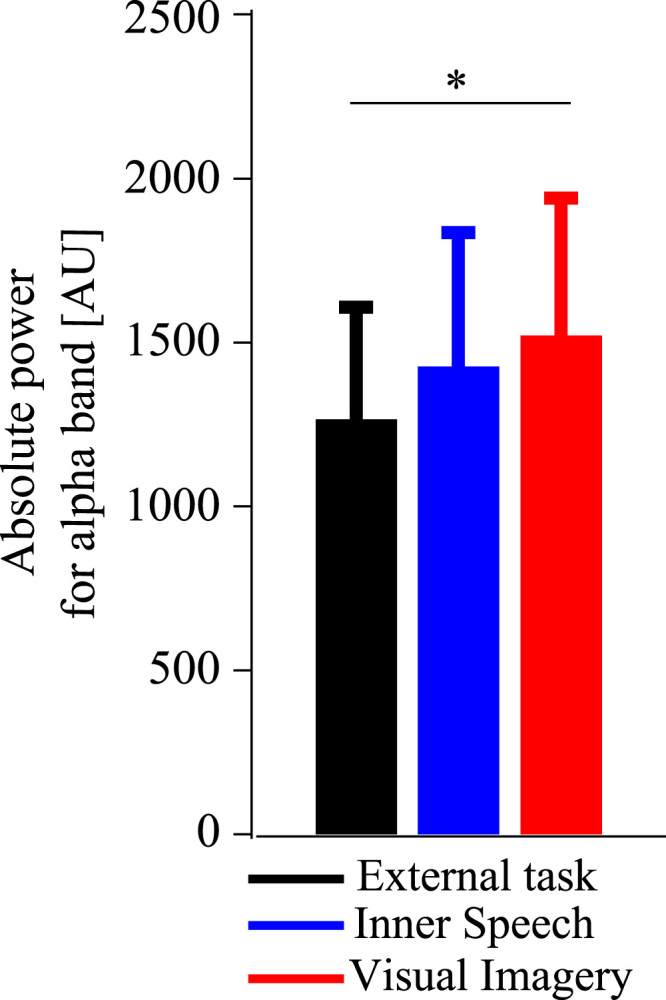
Absolute alpha power for each condition**.** Mean and standard error of absolute power for alpha band. Values were calculated at Parieto-occipital and occipital electrodes for the three conditions. (*p<0.05).

**Fig. 5:**
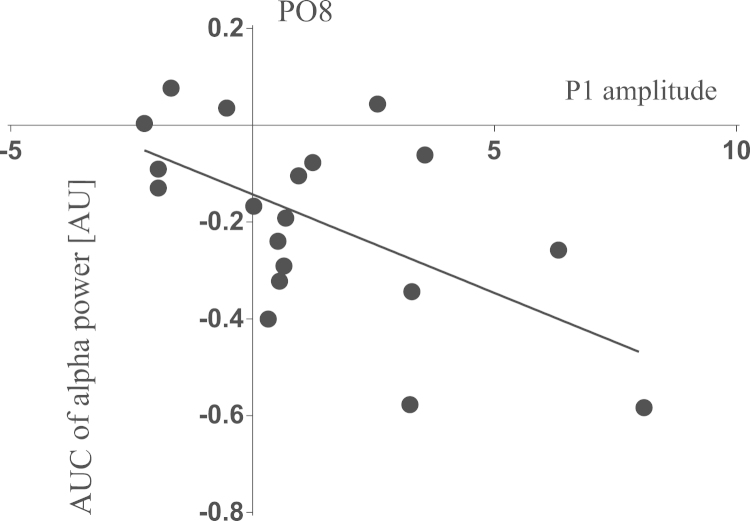
Correlation between P1 amplitude and alpha power modulations**.** Correlation between P1 local peak amplitude difference and AUC alpha power difference at PO8 electrode, when participants performed visual imagery task contrasted with external task.

P1 and N1 amplitudes are shown in Fig. 1, Fig. 2, respectively. Fig. 3 shows the P300 amplitude as well as Fig. 4 shows the absolute alpha power results. Also we show a correlation between P1 local peak amplitude difference (external task – visual imagery) and alpha power difference (external task – visual imagery) at PO8 electrode (Fig. 5).

## 2 Experimental design, materials and methods

### 2.1 Participants

Twenty six volunteers initially participated in this study. Because of excessive ocular/muscular artifacts and problems in data acquisition, six subjects were rejected for further analysis. Thus, the results from 20 subjects (12 women) are reported here (mean age=24.43, range=19–33). Participants gave informed consent and had no history of drug abuse, neurological or psychiatric conditions. The Ethics Committee of Pontificia Universidad Católica de Chile approved the study. All experiments were performed at the Neuro-dynamic Laboratory of the School of Psychology of this University.

### 2.2 Stimuli and procedure

All stimuli were presented on a computer screen. Psychopy software [3] was used to design and present the experiment. The task was composed of 2 blocks (same task used in [1]); each block had 30 auto-administrated trials. Participants started each trail by pressing a button, after this, either the word “imagine” or “speech” came up in the middle of the screen. Next, a red or green, 2 cm radius circle appears indicating whether the attention should be allocated in the external stimuli (red) or in their thoughts (green). After this 10 square (sides were 3 cm) stimuli with a checkerboard pattern reversal appeared sequentially. Each square was presented for 0.2 s.

When the word “imagine” appears followed by the green circle, participants were instructed to think about anything they wanted, given that it had a strictly visual quality, avoiding auditory or phonological elements of any form. This condition will be referred to as: “visual imagery”. If the word “speech” was followed by a green circle participants were instructed to think using their inner speech without using any sort of mental image. This condition will be referred to as: “inner speech”. Finally, if any of the two words were followed by a red circle, participants were instructed to count 10 of the appearing square stimuli and mark the tenth square using the spacebar. This condition will be referred to as: “external task”. Color cues were counterbalanced, i.e. during the second block the red circle cued the thought related tasks and the green circle cued the visual task.

### 2.3 Recordings

EEG data was obtained using 64 electrodes (Biosemi® ActiveTwo) arranged according to the international 10/20 extended system. Horizontal and vertical eye movements were monitored using four external electrodes. Horizontal EOG was recorded bipolarly from the outer canthi of both eyes and vertical EOG was recorded from above and below of the participant׳s right eye. Two additional external electrodes were placed on the right and left mastoid to be used for later re-referencing.

### 2.4 Data pre-processing

Data pre-processing was performed using Matlab 7.8.0 (The Mathworks, Inc.) with EEGLAB v7.1.7.18b toolbox [4]. The signal was down-sampled off-line at 512 Hz for ERP analysis and at 1024 Hz for frequency analysis. Because of hardware setup constraints, all electrodes were referenced to CMS and DRL during acquisition, but off-line re-referenced to averaged mastoids. Horizontal and Vertical EOG were calculated by means of the difference between left-right electrodes and above-below electrodes, respectively. A 2nd order infinite impulse response (IIR) Butterworth filter was used for band-pass filtering continuous EEG data, with a half amplitude cut-off frequency of 0.1 Hz and 30 Hz for ERP analyses and a half amplitude cut-off frequency of 0.1 Hz and 100 Hz for frequency analyses. Artifact detection was performed on segmented data (see below epoch extraction details) using a moving window peak-to-peak threshold algorithm, with a voltage threshold of 100 μV. Manual inspection was also performed in order to detect other artifacts like muscle activity. This was done in a manner that was blind to experimental condition. All detected artifacts were rejected.

### 2.5 ERP analysis

The EEG signal was segmented into 20 trials per conditions, each trial window captured from the appearance of the colored circle up to the trial׳s end. Further segmentation in epochs was applied for each condition, selecting the 200 ms preceding each checkerboard appearance up to 500 ms after that. All of the epochs corresponding to the first appearing checkerboard were discarded given that participants were less likely to have already engaged in a thought process. In the external task condition the epochs corresponding to the last appearing checkerboard were discarded due to possible motor execution artifacts. The average number of epochs per participant was 113.1 (SE: 11.7) for the visual imagery condition, 110.45 (SE: 12.18) epochs for the inner speech condition, and 110.35 (SE: 12.45) epochs for the external task condition.

Epochs were averaged across trials for each subject and condition. The P1 peak was identified as the highest recorded voltage occurring in the 70–140 ms time window following a checkerboard stimulus. The N1 peak was defined as the lowest recorded voltage occurring in the 110–190 ms time window following a checkerboard stimulus. The PO7 and PO8 electrodes were used to find both the P1 and N1 components. The chosen latencies and topographical distributions of early components are described in previous research [5]. We then calculated local peak and mean amplitude for P1 component, N1 component and the peak-to-peak P1/N1 amplitude according to Luck [2], for each subject and condition.

The P300 amplitude was calculated as local peak amplitude in a time windows between 330 and 360 ms at Pz electrode.

### 2.6 Spectral frequency analysis

The following analysis used 1 s epochs free of any stimulus; these epochs were found from the appearance of the colored circle until the trial׳s end for every subject, trial and condition. The average number of epochs per participant was 101.25 (SE: 11.16) for the visual imagery condition, 100.45 (SE: 10.7) epochs for the inner speech condition, and 100.15 (SE: 10.65) epochs for the external task condition. Fourier spectrogram was calculated using an in-house script in MATLAB (The Mathworks, Inc). Occipital and parieto-occipital electrodes showed the greatest alpha power in scalp topography; in particular PO7, PO3, O1, O2, PO4 and PO8 were selected for further analysis.

Spectrogram data were averaged across epochs for each subject for the three conditions. Alpha range was defined as a window of 2 Hz above and 2 Hz below to the maximum power value in the classical alpha range (8–12 Hz) for each subject. Area under the curve (AUC) of alpha power was calculated averaging alpha range for each subject. Relative power was calculated by dividing AUC alpha range by the total AUC [6].

### 2.7 Statistics

Local peak amplitude, mean amplitude and peak-to-peak amplitude of P1/N1 components evoked by attentional checkerboard probes were compared with repeated measures ANOVA using Electrode (two levels: PO7/PO8) and Condition (three levels: visual imagery, inner speech, external task) as factors. Alpha power AUC were compared with repeated measures ANOVA using hemisphere (two levels: right,left), Electrode (three levels: PO7/8,PO3/4,O1/2) and Condition (three levels: visual imagery, inner speech, external task) as factors.

In all cases Mauchly׳s Sphericity Test was carried out, and Greenhouse–Geisser corrections were applied when necessary. In order to determine what conditions differed from each other, further analyses were done by applying an univariate test of significance for planned comparison.

P1 local peak amplitude difference between external task and visual imagery was calculated as well as the AUC alpha power difference between the same conditions. A correlation analysis was performed between both variables at the PO8 electrode. This was carried out on 19 participants due to one of the subject rejected for ERP analysis was not the same rejected for spectral analysis. All statistics were done using STATISTICA 7.0 software (StatSoft, Inc.).

Statistical analysis for P1/N1 peak-to-peak amplitude revealed a main effect for the condition factor, *F* (1.47, 27.96)=4.528, *p*=0.029. A significant difference was found when both representational formats were compared with planned comparison (*F* (1, 19)=5.3, *p*=0.032).

Statistical analysis for local peak P1 amplitude revealed a main effect for the condition factor, *F* (1, 38)=6.503, *p*=0.003. A further analysis was carried out with planned comparisons, revealing a significant difference between external task and inner speech conditions (*F* (1, 19)=4.71, *p*=0.042), but also when both representational formats were compared (*F* (1, 19)=4.98, *p*=0.037) showing a greater attenuation for visual imagery condition.

Statistical analysis for P1 mean amplitude revealed a main effect for the condition factor, *F*(2, 38)=5.887, *p*=0.005. A significant difference was found when both representational formats were compared with planned comparison (*F* (1, 19)=5.823, *p*=0.026).

N1 local peak amplitude shows no differences between conditions (*F* (2, 38)=2.718, *p*=0.078). N1 mean amplitude measurement neither shows differences (*F* (2, 38)=1.48, *p*=0.24).

P300 amplitudes were compared with repeated measures ANOVA using Condition (three levels: visual imagery, inner speech, external task) as factors. An increase in voltage can only be seen in the external task condition *F* (2, 38)=6.931, *p*=0.002.

Statistical analysis for absolute power of alpha band revealed a main effect for the condition factor *F*(2, 38)=4.384, *p*=0.019. A significant difference was found between external task and visual imagery condition (*F* (1, 19)=9.232, *p*=0.006). Also we analyzed absolute power of alpha broadband (8–12 Hz). This result showed a main effect for the condition factor *F*(2, 38)=4.247, *p*=0.021. A significant difference was found between external task and visual imagery condition (*F* (1, 19)=9.08, *p*=0.007).

Correlation was found between P1 local peak attenuation and AUC alpha power enhancement at PO8 electrode, when participants performed visual imagery task contrasted with external task (*r*^2^=0.337, *p*=0.009).
